# Incidentally diagnosed melorheostosis of upper limb: case report

**DOI:** 10.1186/s12891-015-0455-z

**Published:** 2015-01-31

**Authors:** Vaclav Vyskocil, Karel Koudela, Tomas Pavelka, Kristyna Stajdlova, David Suchy

**Affiliations:** Department of Orthopaedic Surgery, Faculty Hospital Plzen, Alej Svobody 80, Plzen, 304 60 Czech Republic; Department of Imaging Methods, Charles University Teaching Hospital Plzen and Medical Faculty Plzen, Alej Svobody 80, Plzen, 304 60 Czech Republic; Department of Clinical Pharmacology, Charles University Teaching Hospital E. Benese 13, Plzen, 30599 Czech Republic

**Keywords:** Melorheostosis, Melting wax, Joint contracture, Swelling, Osteopoikilotic islands, Bisphosphonates, COX2-inhibitors

## Abstract

**Background:**

Melorheostosis is quite a rare bone disease with still unclear ethiology. Although multifocal affection is highly debilitating with unfavorable prognosis, there is no clear consensus about therapeutical approach. There is still insufficient evidence in the literature for almost a century after the first description.

Affected bone has a typical appearance of melting wax. Diagnosis is usually incidental with pain as a leading symptom. Diagnosis itself is relatively easy, routine X-ray examination is sufficient. Even though it could be easily overlooked and mistaken with other diseases. Melorheostosis is incurable, the therapy is mostly focused on maintaining patient quality of life.

Presented case is unique in terms of extent of the affection (index finger, metacarp shaft, carpal bones, forearm, humerus and whole scapula) in combination with osteopoikilotic islands in other 3 regions (vertebrae, manubrium sterni and left collar bone). Currently there is only one such a case published in the literature (Campbell), but without osteopoikilotic islands.

**Case presentation:**

Melorheostosis was diagnosed in 26-year old female after injury as an incidental finding. This was quite surprising as the patient already suffered by limited movement in the upper limb and pain before the injury. Detailed examination were performed to confirm the diagnosis, no family history was found. Pharmacotherapy with bisphosphonates, non-steroidal antirheumatics and vasodilatans/rheologic drugs seemed to be effective to maintain the relatively good quality of patient life and good performance in daily routine. Questionable is further development of patient performance status and sustainability of conservative treatment in the long term follow up.

**Conclusion:**

Conservative treatment with bisphopshonates and COX-2 inhibitors in combination with naftidrofuryl can delay surgery solution.

## Background

Melorheostosis is a rare bone disease. Only about 300 cases have been reported worldwide [1]. The etiology is still unknown. It is a developmental anomaly of bone formation with the evidence of inheritance. The small part of patients have mutations in LEMD3 group, but this mutations was not present in most of the cases.

This anomaly was firstly described in 1922 by Leri and Joany [2]. Typical for melorheostosis is a presence of bone sclerosis with a linear pattern mainly affecting the cortex of tubular bones which is identifiable by plain radiography [3]. Melorheostosis can occur at any age and both sexes are affected equally.

The linear hyperostosis of cortex can extend and affect medullar canal and periosteum resembling a typical “melting wax” appearance of the affected bone [4]. Hyperostosis is very often accompanied by hyperplasia and abnormalities of adjacent connective tissues [5]. Melorheostosis may be asymptomatic for a long time but often leads to joint contractures, swelling, stiffness and chronic pain [6]. Peak age of diagnosis is between 5 and 20 years [7].

Besides radiographic changes melorheostosis has 9 typical signs [8]: thickening of outer layer of bone, skin affection, intermittent joint swelling, joint pain, limb deformity, nerve oppression, pain, paresthesia and reduced range of motion. Other associated tissues are dermal and soft tissue lesions such as linear scleroderma, vascular malformations, hemangioma, neurofibromatosis, arterial aneurysm, tuberous sclerosis and focal subcutaneous fibrosis [9].

There are 2 theories for melorheostosis etiology: (a) early embryonic infection of a sensory nerve inducing the changes in sclerotome [10] and (b) concept of “mosaicism” which can better explain an asymmetric segmental pattern with variable expressivity and equal gender ratio of the disease [11].

## Case presentation

26-year old female patient underwent surgery of a cleft palate at the age of 5 years, later she had no significant health issue. Melorheostosis was diagnosed incidentally by X-ray after shoulder injury in sport.

Clinical examination showed reduced joint abduction 60 degress in the shoulder, further movement was possible only in scapula, movement range in the elbow was limited to 20 degrees, in radiocarpal joint was volar flexion up to 40 degrees, dorsal flexion 0, pronation and supination was also 0. The patient reported pain in the whole upper limb at rest. Osteopoikilotic islands were identified in the body of third cervical vertebra and also in the right collar bone and sternum manubrium. CT of the upper limbs and upper chest was performed without contrast tracer. There was no family history of melorheostosis found.

**Figure 1:**
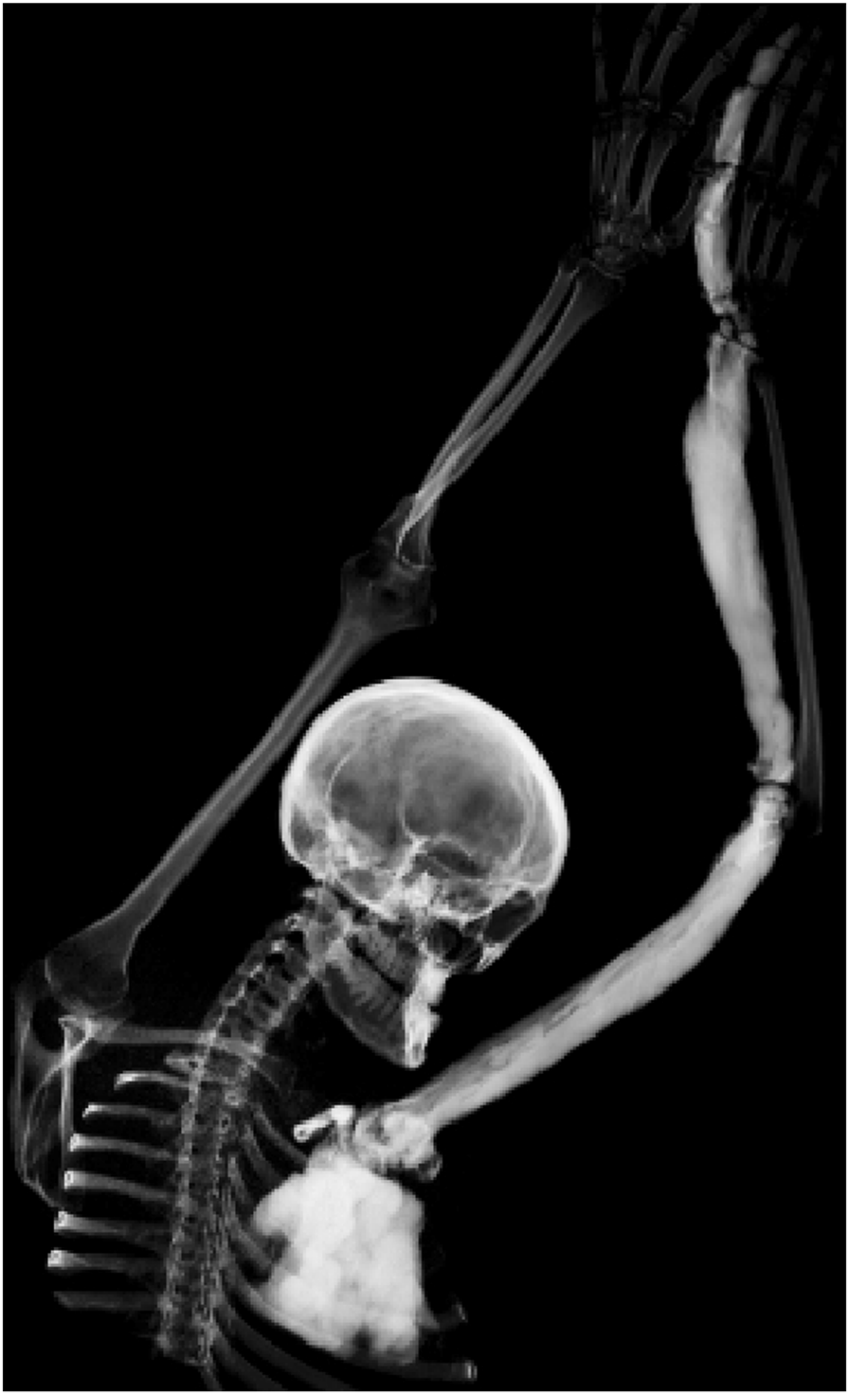
**CT of left hand and forearm.** Diffuse thickening and sclerotisation on index finger and II. metacarp shaft. The lesion is less aparent at the I. metacarp and on some carpal bones. There is diffusely enlarged sclerotic radius and humerus without ulna bridging. Whole scapula on the left side is also affected. The cavities on the affected bones are completely filled with the sclerotic bone.

**Figure 2:**
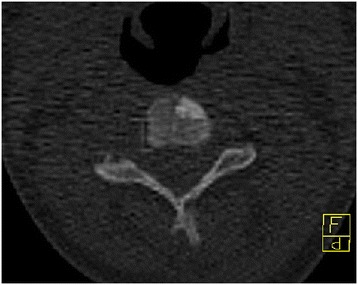
**CT of cervical spine.** Small sclerotic focus is located in the ventral part of C3 vertebral body - size 7 mm.

Diffuse thickening and sclerotisation was identified on index finger, II. metacarp shaft on the left hand, the lesion was less aparent at the I. metacarp of the left hand and on some carpal bones (Figure 1). There was diffusely enlarged sclerotic radius, humerus and the scapula on the left side. The cavities on affected bones were completely filled with the sclerotic bone. Small sclerotic focus was in the ventral part of C3 vertebral body - size 7 mm (Figure 2), there was also a small island in the left manubrium sternum and in the sternal part of the left collarbone. The finding corresponded to melorheostosis Léri. Thickness of patient’s left scapula was up to 31 mm, the contralateral scapula was unaffected with thickness of 2–3 mm. The cortex of both collarbone and ribs had slightly higher bone density. The skull was without pathological finding.

**Figure 3:**
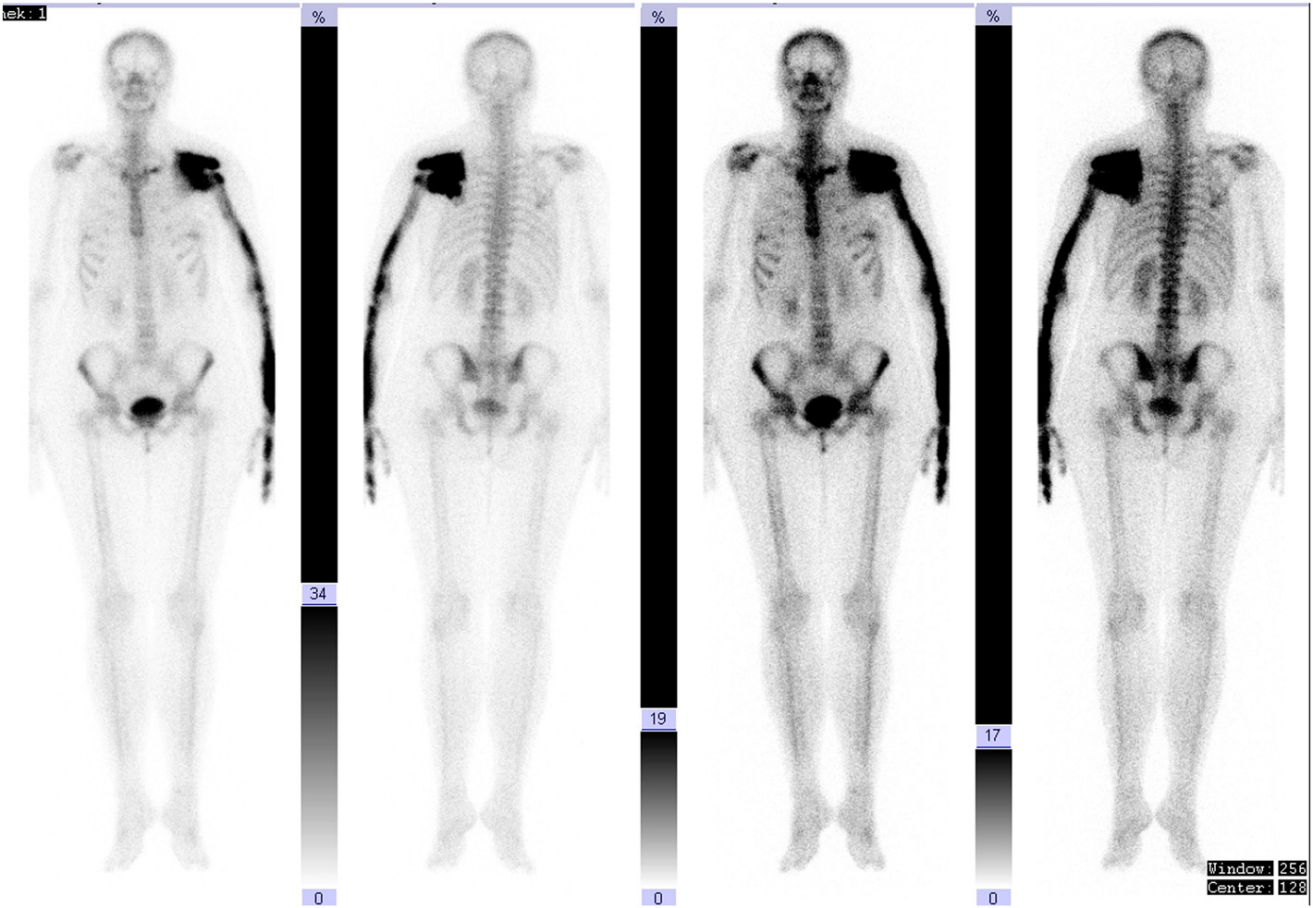
**Whole body bone scintigraphy.** Significant, locally inhomogeneous increase of activity is evident in most of the scapula, humerus, radius and in II. shaft on the left, the highest intensity of changes are in the scapula, which shows 7-times higher activity compared to the contra lateral parts. Activity increase in the I. shaft left is more modest and slight accumulation is present in the left medial clavicle and manubrium sterni.

Bone scintigraphy showed normal level of overall metabolic activity in the skeleton. Significant, locally inhomogeneous increase of activity was evident in most of the scapula, humerus, radius and in II. shaft on the left, the highest intensity of changes was in the scapula, which showed 7-times higher activity compared to the contra lateral parts. Activity increase in the I. shaft left was more modest and slight accumulation was present in the left medial clavicle (Figure 3). The rest of skeleton was without significant pathological changes.

DXA examination of the whole skeleton showed osteopenia in the lumbar spine: T score −1.2 SD, in both hips T-score was same and within the normal range. There was no abnormality found in the laboratory tests including bone markers except for increased level of osteocalcin and C-terminal telopeptide.

The published cases reported good experience with bisphosphonate in terms of disease progression [12]. The patient was treated with weekly alendronate and COX-2 inhibitor (celecoxib). In connection with the development of sleep disorders and increased skin sensitivity sensitivity on affected site naftidrofuryl in high doses (600 mg daily) was added, which led to symptoms relieve. The patient is currently without pain and is able to perform normal daily activities with non-progressive restriction for 5 years after the diagnosis. Pharmacological treatment in described combination could delay surgery solution and eventually could prevent an excessive dosage of analgetics. Questionable is further development of patient performance status and sustainability of conservative treatment in the long term follow up.

## Conclusion

X-ray is a sufficient method for diagnosis of melorheostosis. Other imaging techniques are essential for decision about therapeutic intervention (CT, MRI, scintigraphy and DXA). Laboratory findings are usually in physiological range (calcium, phosphorus, markers of bone formation and resorption, IGF-1). Symptomatic therapy proved to be sufficient in subjective symptoms management. The long term effect of conservative treatment remains questionable.

## Consent

Written informed consent was obtained from the patient for publication of this Case report and any accompanying images. A copy of the written consent is available for review by the Editor of this journal.

## Abbreviation

CT: Computer tomography
MRI: Magnetic resonance imaging
DXA: Dual X-ray absorptionmetry
SD: Standard deviation
